# Neuroimmune regulation of Alzheimer’s pathology by age‐related blood‐borne factors

**DOI:** 10.1002/alz.083657

**Published:** 2025-01-03

**Authors:** Brittany M. Hemmer, Alejandro B. Grau‐Perales, Ana Catarina Ferreira, Ralphyn Pallikunnath, Jacob L. Rosenstadt, Joseph M. Castellano

**Affiliations:** ^1^ Icahn School of Medicine at Mount Sinai, New York, NY USA; ^2^ Icahn School of Medicine at Mount Sinai, Ronald M. Loeb Center for Alzheimer’s disease, Nash Family Department of Neuroscience, Friedman Brain Institute, New York, NY USA

## Abstract

**Background:**

Alzheimer’s disease (AD) is a complex neurodegenerative disorder characterized by hallmark pathologies that affect many brain regions, including the cellular microenvironment with the hippocampus, ultimately leading to profound deficits in cognition. Surprising recent work has shown that factors in the systemic environment regulate the hippocampal cellular niche; age‐associated blood‐borne factors exacerbate brain aging phenotypes, whereas youth‐associated blood‐borne factors, including tissue inhibitor of metalloproteinases 2 (TIMP2), reverse or ameliorate features of brain aging. As aging serves as the major risk factor for AD, and recent work shows that systemic factors can regulate AD pathology, we sought to characterize mechanisms by which the systemic environment regulates CNS phenotypes relevant to AD pathology through changes in neuroinflammation.

**Method:**

We used both global and conditional deletion approaches in wildtype and APP‐knockin (NL‐F) or 5XFAD mice to target sources of TIMP2, a youth‐associated systemic factor previously shown to rejuvenate the aged hippocampus. We applied conventional and super‐resolution confocal microscopy approaches to characterize the impact on amyloid pathology and neuroinflammation. Changes in microglial morphology and state in the setting of TIMP2 modulation were characterized using a variety of approaches, including in vivo microdialysis and snRNA‐sequencing. We also restored levels of TIMP2 in aged APP‐KI mice to examine the impact on cognitive deficits. Conversely, we systemically injected APP‐KI mice with proteins associated with advanced age to examine the impact of age‐accumulating blood‐borne factors on amyloid accumulation and microglial state.

**Result:**

Deletion of TIMP2 from specific sources is associated with significant changes in microglial state. Its loss is associated with dysfunctional activation state, senescence, and perturbed morphology. We also find that TIMP2 deletion exacerbates accumulation of amyloid in various brain regions associated with accumulated extracellular matrix and its rescue in aged APP‐KI mice improves memory. Conversely, injecting specific factors present in aged blood significantly exacerbates amyloid pathology and alters microglial state while impairing adult neurogenesis in the hippocampus.

**Conclusion:**

Together our results highlight mechanisms by which both youth‐associated and age‐associated blood‐borne factors regulate AD pathology. Factors in the systemic environment appear to interact with microglia, cells that are intimately involved in modulation of canonical AD pathology.

